# Prognosis of PCI in AMI setting in the elderly population: Outcomes from the multicenter prospective e‐ULTIMASTER registry

**DOI:** 10.1002/clc.23902

**Published:** 2022-09-07

**Authors:** Majdi Saada, Ofer Kobo, Jawed Polad, Majdi Halabi, Alexander J. J. IJsselmuiden, Ángel Puentes, Jacques Monségu, David Austin, Ruslan K. Baisebenov, Fabrizio Spanó, Ariel Roguin, e‐ULTIMASTER Investigators

**Affiliations:** ^1^ Department of Cardiology, Hillel Yaffe Medical Center Technion—Faculty of Medicine Hadera Israel; ^2^ Department of Cardiology Jeroen Bosch Ziekenhuis 's Hertogenbosch The Netherlands; ^3^ Department of Cardiology Ziv Hospital Safed Israel; ^4^ Cardiology Department Amphia Hospital Breda Breda The Netherlands; ^5^ Department of Cardiology San Juan de Dios Hospital Santiago Chile; ^6^ Department of Cardiology, Groupe Hospitalier Mutualiste Institut Cardiovasculaire Grenoble France; ^7^ Department of Cardiology The James Cook University Hospital Middlesbrough UK; ^8^ Department of Cardiology Regional Cardiology Center Pavlodar Kazakhstan; ^9^ Department of Cardiology Meander Medical Center Amersfoort The Netherlands

**Keywords:** age, coronary artery disease, elderly, myocardial infarction, outcome, stent

## Abstract

**Background:**

Elderly patients with ST‐elevation myocardial infarction (STEMI) who undergo percutaneous coronary intervention (PCI) are usually excluded from major trials.

**Hyopthesis:**

This study sought to assess 1‐year clinical outcomes following PCI with a drug‐eluting stent in patients older than 80 years old with STEMI.

**Methods:**

The large all‐comer, multicontinental e‐ULTIMASTER registry included 7507 patients with STEMI who underwent PCI using the Ultimaster stent. The primary clinical endpoint was 1‐year target lesion failure, a composite of cardiac death (CD), target vessel‐related myocardial infarction (TV‐MI), or clinically driven target lesion revascularization (CD‐TLR).

**Results:**

There were 457 (6.1%) patients in the elderly group (≥80 years old) that were compared to 7050 (93.9%) patients <80 years. The elderly patients included more female patients and had significantly more comorbidities and had more complex coronary anatomy. The primary endpoint occurred in 7.2% of the elderly, compared to 3.1% of the younger group (*p* < .001). All‐cause mortality was significantly higher among the elderly group compared to the younger group (10.1% vs. 2.3%, *p* < .0001), as well as CD (6.1% vs. 1.6%, *p* < .0001), but not TV‐MI (1.1% vs. 0.7%, *p* = .34) or CD‐TLR (1.1% vs. 1.4%, *p* = .63).

**Conclusion:**

Elderly patients with STEMI presentation had a higher incidence of the composite endpoint than younger patients. All‐cause and CD were higher for elderly patients compared to patients younger than 80 years old. However, there was no difference in the incidence of TV‐MI or target lesion revascularizations. These findings suggest that PCI for STEMI in elderly patients is relatively safe.

**Abbreviations**
CABG: coronary artery bypass grafting
CAD: coronary artery disease
CD‐TLR: clinically driven target lesion revascularization
CI: confidence interval
DES: drug‐eluting stent
IPSW: inverse propensity score weighting
MI: myocardial infarction
OR: odds ratio
PCI: percutaneous coronary intervention
POCE: patient‐oriented composite endpoint
STEMI: ST‐elevation myocardial infarction
TLF: target lesion failure
TV‐MI: target vessel‐related myocardial infarction

## 1 INTRODUCTION

Coronary artery disease (CAD) is the most common cause of death worldwide and is a leading cause of mortality and morbidity in the elderly.^1^, ^2^, ^3^, ^4^ The population all around the world is aging, in part thanks to the progress made in medical science. The number of elderly patients with acute myocardial infarction (AMI) undergoing percutaneous coronary intervention (PCI) has also increased in recent years^5^, ^6^, ^7^ and is expected to grow even further.^8^

Older studies have suggested that age is an independent predictor of adverse outcomes following PCI.^9^, ^10^ Elderly patients (age ≥ 80) are often excluded from major clinical trials of cardiovascular interventions because of concerns about the increased risk of adverse events and limited life expectancy.^11^ In addition, older patients with CAD are less likely than younger patients to undergo invasive revascularization even in current clinical practice.^12^, ^13^, ^14^ In the acute setting, despite current evidence and recommendations, older patients are still less likely to receive reperfusion treatment when compared with their younger counterparts. Efforts should be made to improve this picture, as invasive strategies in ST‐elevation myocardial infarction (STEMI) associate greater survival in elderly patients, and there is no upper age limit for urgent reperfusion.^15^, ^16^

Therefore, knowledge regarding the outcome of the elderly referred for PCI in the current era of improved techniques, devices, and pharmacotherapy is limited. Prior studies have shown clear clinical benefits when performing PCI over medical therapy in the elderly group presenting with STEMI.^17^, ^18^, ^19^, ^20^, ^21^ Nonetheless, the long‐term outcome of the elderly population compared to the younger one is not well established. The purpose of the current study is to investigate the characteristics and clinical outcomes in the elderly with STEMI presentation undergoing PCI in the contemporary drug‐eluting stent (DES) era from one of the largest cohorts in the world.

## 2 METHODS

The e‐ULTIMASTER registry enrolled 37, 198 patients with an indication for PCI. Very few inclusion and exclusion criteria were applied to evaluate the Ultimaster stent outcomes in an all‐comer patient population.^22^ We analyzed the population according to STEMI presentation and compared the elderly patients (≥80 years) to the nonelderly (<80 years) patients. Our population includes 7507 patients with STEMI. Sites in Europe, Asia, Africa, the Middle East, South America, and Mexico participated in the registry, using a thin‐strut (80 μm) cobalt–chromium sirolimus‐eluting stent. This stent features a biodegradable polymer coating (poly‐d,l‐lactic acid polycaprolactone) that is fully metabolized through dl‐lactide and caprolactone into carbon dioxide and water in 3–4 months. This coating is applied on the abluminal side of the struts only; after resorption, a bare‐metal stent (BMS) remains.

The primary endpoint was 1‐year target lesion failure (TLF) defined as the composite of cardiac death, target vessel‐related myocardial infarction (TV‐MI), and clinically driven target lesion revascularization (CD‐TLR). Patient‐oriented composite endpoint (POCE) was defined as all death, any MI, and any revascularization. For myocardial infarction (MI), the extended historical MI definition was applied which primarily uses creatine kinase myocardial band, or if not available troponin, as cardiac biomarker criterion. All primary endpoint‐related events were adjudicated by an independent clinical events committee. The study was approved by the ethical committees of the participating sites and all patients provided written informed consent. The clinicaltrial.gov identifier is NCT02188355.

Follow‐up has been performed 3 months and 1‐year postindex procedure. Patients were contacted by telephone or by a visit to the outpatient clinic. Relevant information on adverse events, that is, death, MI, re‐PCI, coronary artery bypass grafting (CABG), bleeding, vascular complication, or stent thrombosis were collected.

### 2.1 Statistical analysis

**Figure A1:**
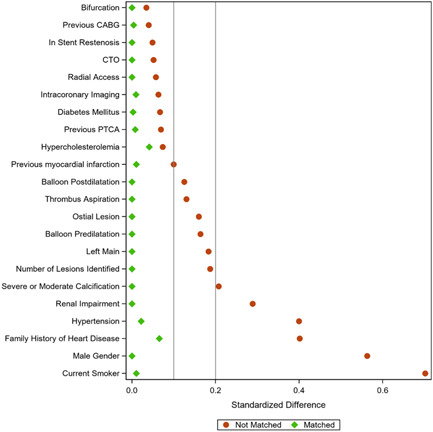
Standardized differences in variables included in the propensity score between groups. After adjustment, all covariates in the planned propensity score had weighted standardized differences below 0.1, which indicates an equilibration of these covariates between the groups. CABG, coronary artery bypass grafting; CTO, chronic total occlusion; PTCA, percutaneous transluminal coronary angioplasty.

Continuous variables are presented as the mean ± standard deviation and compared using the Student's *t*‐test. Categorial variables are presented as frequencies (percentage) and compared using the *χ* ^2^ test or Fischer's exact test, as appropriate. The cumulative events rates were estimated by the Kaplan–Meir method and compared by the logrank test. Hazard ratios and 95% confidence intervals (CIs) were calculated with Cox hazards regression analysis. An inverse propensity score weighting (IPSW) analysis was performed to address differences in baseline patient and lesion characteristics including 22 variables: current smoker, male gender, renal impairment, family history of heart disease, hypertension, severe/moderate calcification, number of lesions identified, balloon predilatation, left main, previous CABG, thrombus aspiration, previous percutaneous transluminal coronary angioplasty, ostial lesion, bifurcation lesion, chronic total occlusion, intravascular imaging, hypercholesterolemia, in‐stent restenosis, acute coronary syndrome (ACS), diabetes mellitus, balloon postdilatation, and radial arterial access (Figure A1). After adjustment, all covariates in the planned propensity score had weighted standardized differences below 0.1, indicating an equilibration of these covariates between the groups. To identify predictors of TLF at 1 year, a stepwise logistic regression model including the above‐mentioned variables was performed applying *p*‐values set to *p* = .25 and *p* = .10 to enter and to stay in the model, respectively. *p* < .05 were considered statistically significant. Analyses were performed with SAS software, version 9.4 (SAS Institute Inc.).

## 3 RESULTS

**Figure A2:**
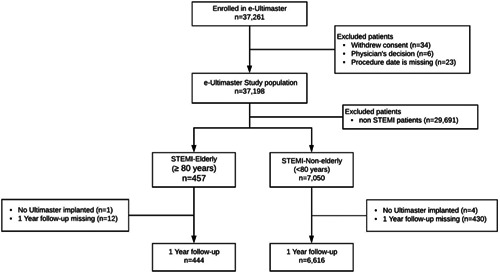
Patient disposition of patients enrolled in the e‐ULTIMASTER study and grouped according to STEMI‐elderly (≥80 years) and STEMI‐nonelderly (<80 years). STEMI, ST‐elevation myocardial infarction.

A total of 7507 STEMI patients who underwent PCI were included in our analysis (Figure A2), of which 457 (6.1%) were in the elderly group. Baseline characteristics of these patients as well as procedural characteristics are shown in Table 1. The mean age of the elderly group was 83.6 ± 3.5 years old, compared to 59.4 ± 10.4 years of age in the younger group (*p* < .0001). Patients in the elderly group were more likely to be females (47.5% vs. 19.8%, *p* < .0001), and to have more hypertension and renal disease compared to the younger patients (74.2% vs. 54.7% and 11.5% vs. 3.6%, respectively, both *p* < .0001), while they were less smokers (9.1% vs. 43.7%, *p* < .0001).

**Table 1:** Baseline patient and angiographic characteristics

	STEMI elderly (≥80 years) *n* = 457	STEMI nonelderly (<80 years) *n*= 7050	*p*‐Value
Age (years), mean ± SD	83.6 ± 3.5 (457)	59.4 ± 10.4 (7050)	<.0001
Male gender	52.5 (240/457)	80.2 (5657/7050)	<.0001
Diabetes mellitus	23.9 (107/447)	21.1 (1466/6939)	.16
Insulin‐dependent diabetes mellitus	3.6 (16447)	4.1 (284/6939)	.59
Hypertension	74.2 (311/419)	54.7 (3468/6339)	<.0001
Hypercholesterolemia	44.6 (175/392)	48.3 (2881/5964)	.16
Current smoker	9.1 (32/352)	43.7 (2534/5803)	<.0001
Peripheral vascular disease	6.4 (27/420)	3.0 (190/6448)	<.0001
Renal impairment	11.5 (51444)	3.6 (250/6899)	<.0001
Previous myocardial infarction	12.6 (54/429)	9.5 (621/6570)	.03
Previous PTCA	10.2 (44/431)	8.2 (541/6590)	.15
Previous CABG	1.9 (8/430)	1.4 (89/6554)	.39
Number of lesions treated at index procedure, mean ± SD	1.3 ± 0.6 (457)	1.2 ± 0.5 (7039)	<.0001
Coronary arteries treated per patient			
Left main	4.4 (20/457)	1.3 (93/7050)	<.0001
Right coronary artery	38.1 (174/457)	38.6 (2721/7050)	.82
Left anterior descendants	49.9 (228/457)	49.8 (3512/7050)	.98
Left circumflex	20.1 (92/457)	19.7 (1387/7050)	.81
Arterial or venous graft	0.4 (2/457)	0.4 (25/7050)	.77
Any chronic total occlusion	1.3 (6/457)	2.0 (139/7050)	.32
Any bifurcation	8.3 (38/457)	7.4 (521/7050)	.47
Any long lesion (stent length ≥25 mm)	32.0 (146/457)	37.4 (2636/7050)	.02
Any small vessel (stent diameter ≤ 2.75 mm)	44.2 (202/457)	33.0 (2325/7050)	<.0001
Radial access	86.9 (397/457)	83.3 (5869/7050)	.04
Intracoronary imaging	4.6 (21/457)	3.3 (231/7050)	.13
Ostial lesion (<3 mm) per lesion	7.2 (43/594)	4.3 (362/8498)	<.001
Any moderate or severe calcification per lesion	18.5 (110/594)	11.3 (964/8498)	<.0001
Number of stents successfully implanted	1.5 ± 0.9 (457)	1.4 ± 0.7 (7000)	<.0001
The total length of stents successfully implanted per lesion	24.8 ± 12.4 (538)	26.3 ± 12.9 (7860)	.20
The total length of stents successfully implanted per patient	29.3 ± 17.8 (456)	29.6 ± 16.7 (6987)	.06

Abbreviations: CABG, coronary artery bypass grafting; PTCA, percutaneous transluminal coronary angioplasty; STEMI, ST‐elevation myocardial infarction.

The angiographic characteristics were different between the groups. The elderly group had more lesions treated during the index procedure compared to the younger patients (1.3 ± 0.6 vs. 1.2 ± 0.5, *p* < .0001), with higher rates of the left main coronary artery treatment (4.4% vs. 1.3%, *p* < .0001), calcified lesions (18.5% vs. 11.3%, *p* < .0001), ostial lesions (7.2% vs. 4.3%, *p* < .001), and number of stents implanted (1.5 ± 0.9 vs. 1.4 ± 0.7, *p* < .0001). The radial artery approach was used more in the elderly group (86.9% vs. 83.3%, *p* = .04).

**Figure 1:**
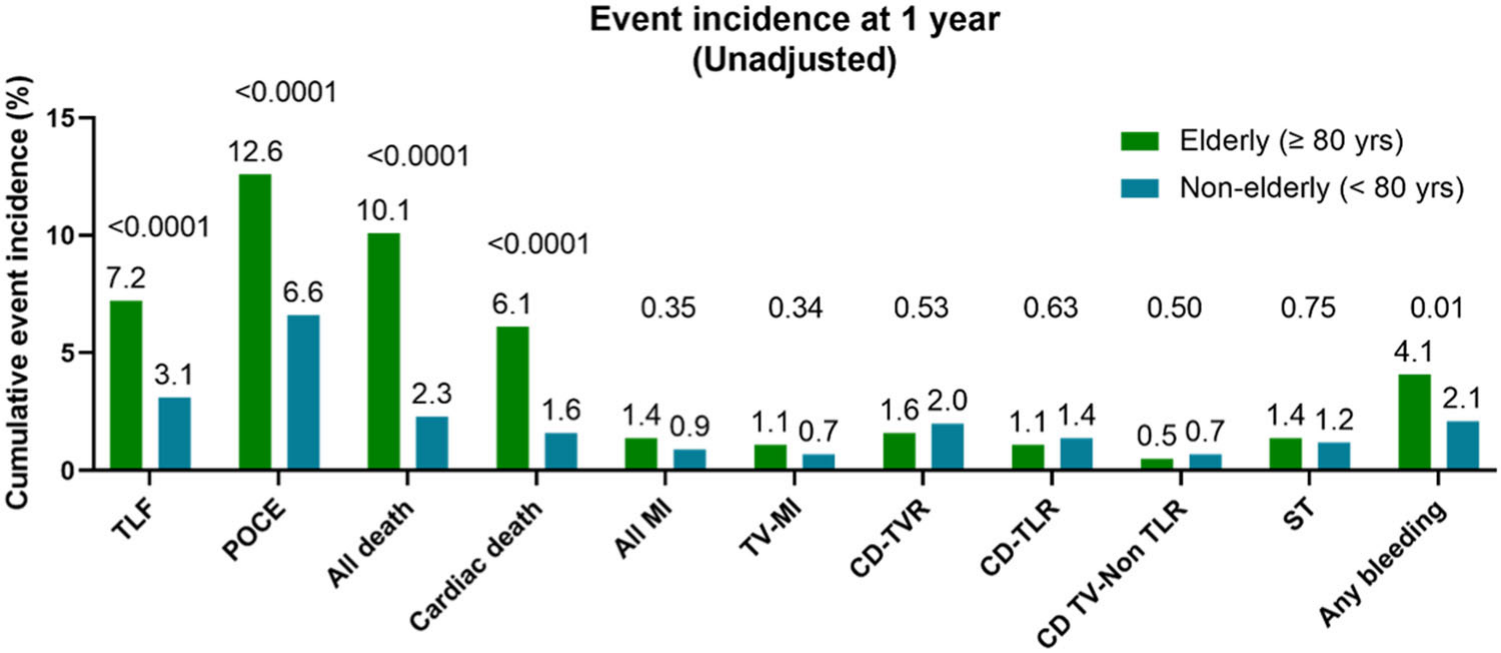
Unadjusted event rate at 1 year. CD, clinically driven; MI, myocardial infarction; POCE, patient‐oriented composite endpoint; ST, stent thrombosis (definite/probable); TLF, target lesion failure; TLR, target lesion revascularization; TVR, target vessel revascularization; TV‐MI, target vessel myocardial infarction.

There were 7060 (94.0%) patients with 1‐year follow‐up (Figure A2). The crude event rates at 1 year are shown in Table 2 and Figure 1. The primary endpoint of TLF occurred in 7.2% in the elderly group compared to 3.1% in the younger group (*p* < .0001). All‐cause mortality was significantly higher among the elderly group compared to the younger group (10.1% vs. 2.3%, *p* < .0001), as well as death from cardiac causes (6.1% vs. 1.6%, *p* < .0001), POCE (12.6% vs. 6.6%, *p* < .0001), and bleeding (4.1% vs. 2.1%, *p* < .001). There was no significant differences in the rates of all MI (1.4% vs. 0.9%, *p* = .35), TV‐MI (1.1% vs. 0.7%, *p* = .34), CD‐TLR (1.1% vs. 1.4%, *p* = .63), or stent thrombosis (1.4% vs. 1.2%, *p* = .75). At 1 year, 66.4% (261/393) in the elderly group were taking dual antiplatelet therapy and 73.2% (4717/6442) in the nonelderly group, *p* = .23. More patients were using oral anticoagulation in the elderly group: 8.7% (34/393) versus 3.7% (237/6442), *p* < .0001.

**Table 2:** Unadjusted event rates and event rates after inverse‐weighted propensity score adjustment

	Unadjusted analysis			Adjusted analysis		
	STEMI elderly (≥80 years) *n* = 444	STEMI nonelderly (<80 years) *n* = 6616	*p*‐Value	STEMI elderly (≥80 years) *n*= 444	STEMI nonelderly (<80 years) *n*= 6616	*p*‐Value
TLF	7.2 (32/444)	3.1 (202/6616)	<0.0001	7.1% (31/444)	3.8% (252/6616)	<0.001
POCE	12.6 (56/444)	6.6 (437/6616)	<0.0001	12.6% (56/444)	8.3% (549/6616)	<0.01
All death	10.1 (45/444)	2.3 (150/6616)	<0.0001	10.1% (45/444)	3.6% (241/6616)	<0.0001
Cardiac death	6.1 (27/444)	1.6 (108/6616)	<0.0001	6.0% (26/444)	2.4% (159/6616)	<0.0001
All myocardial infarction	1.4 (6/444)	0.9 (60/6616)	0.35	1.4% (6/444)	0.8% (52/6616)	0.17
Target vessel myocardial infarction	1.1 (5/444)	0.7 (48/6616)	0.34	1.2% (5/444)	0.7% (45/6616)	0.26
CD‐TVR	1.6 (7/444)	2.0 (133/6616)	0.53	1.6% (7/444)	2.0% (135/6616)	0.51
CD‐TLR	1.1 (5/444)	1.4 (93/6616)	0.63	1.2% (5/444)	1.5% (97/6616)	0.61
CD‐TV non‐TLR	0.5 (2/444)	0.7 (48/6616)	0.50	0.4% (2/444)	0.7% (45/6616)	0.51
Stent thrombosis, definite and probable	1.4 (6/444)	1.2 (78/6616)	0.75	1.4% (6/444)	1.2% (77/6616)	0.72
Bleeding	4.1% (18/444)	2.1% (141/6616)	0.01	5.2% (23/444)	2.4% (161/6616)	<0.01

Abbreviations: CD, clinically driven; POCE, patient‐oriented composite endpoint; STEMI, ST‐elevation myocardial infarction; TLF, target lesion failure; TLR, target lesion revascularization; TV, target vessel; TVR, target vessel revascularization.

### 3.1 Regression analysis

**Figure 2:**
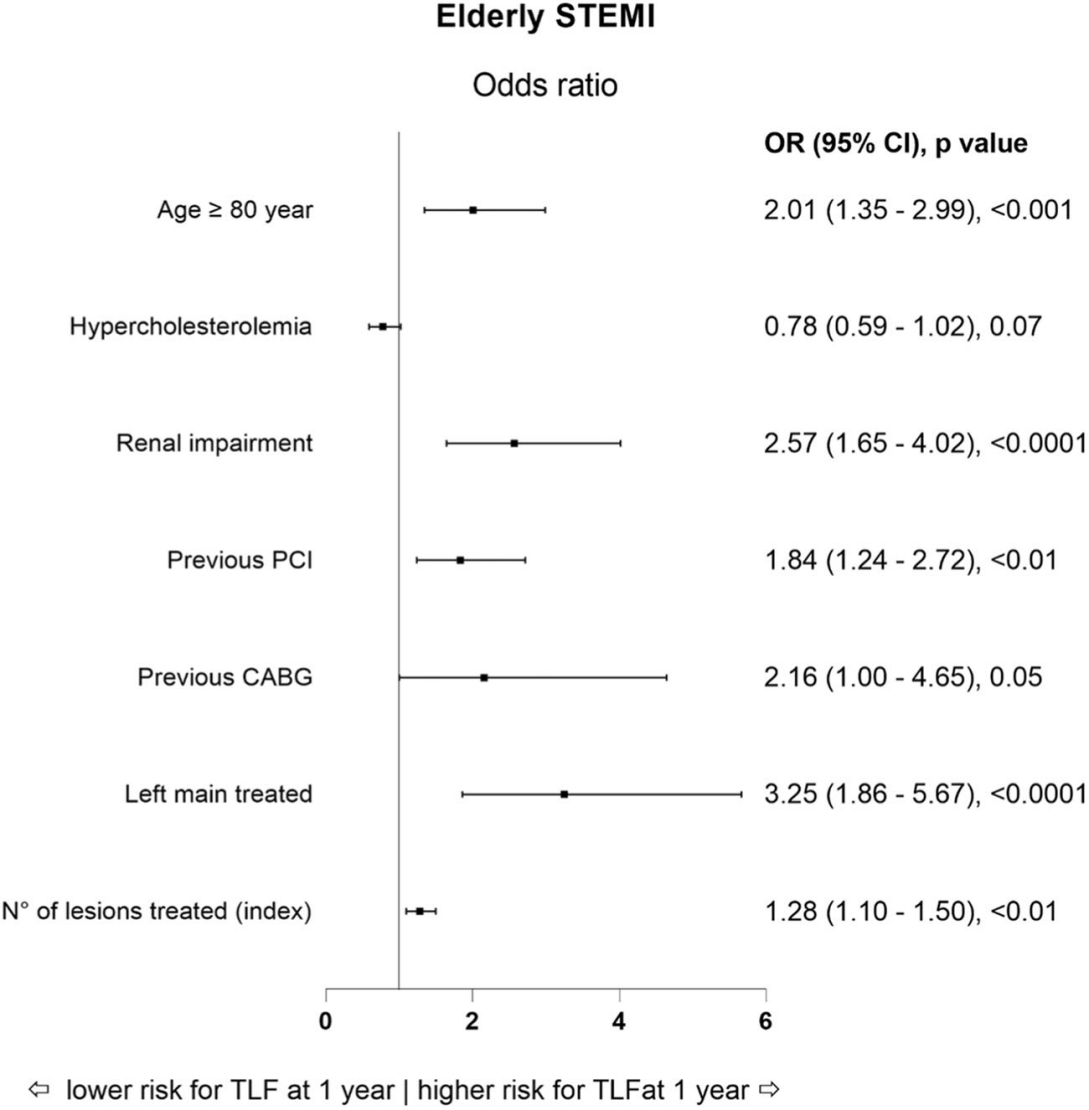
Odds ratio (OR) for TLF rate at 1 year after stepwise multivariable analysis. CABG, coronary artery bypass grafting; CI, confidence interval; PCI, percutaneous coronary intervention; STEMI, ST‐elevation myocardial infarction; TLF, target lesion failure.

In the stepwise regression analysis, age ≥80 years (odds ratio [OR]: 2.01, 95% CI: 1.35–2.99, *p* < .001), renal impairment (OR: 2.57, 95% CI: 1.65–4.02, *p* < .0001), previous PCI (OR: 1.84, 95% CI: 1.24–2.72, *p* < .01), left main treatment (OR: 3.25, 95% CI: 1.86–5.67, *p* < .0001), and the number of lesions treated (OR: 1.28, 95% CI: 1.10–1.50, *p* < .01) emerged as independent predictors for TLF at 1 year (Figure 2).

### 3.2 Propensity analysis

**Figure 3:**
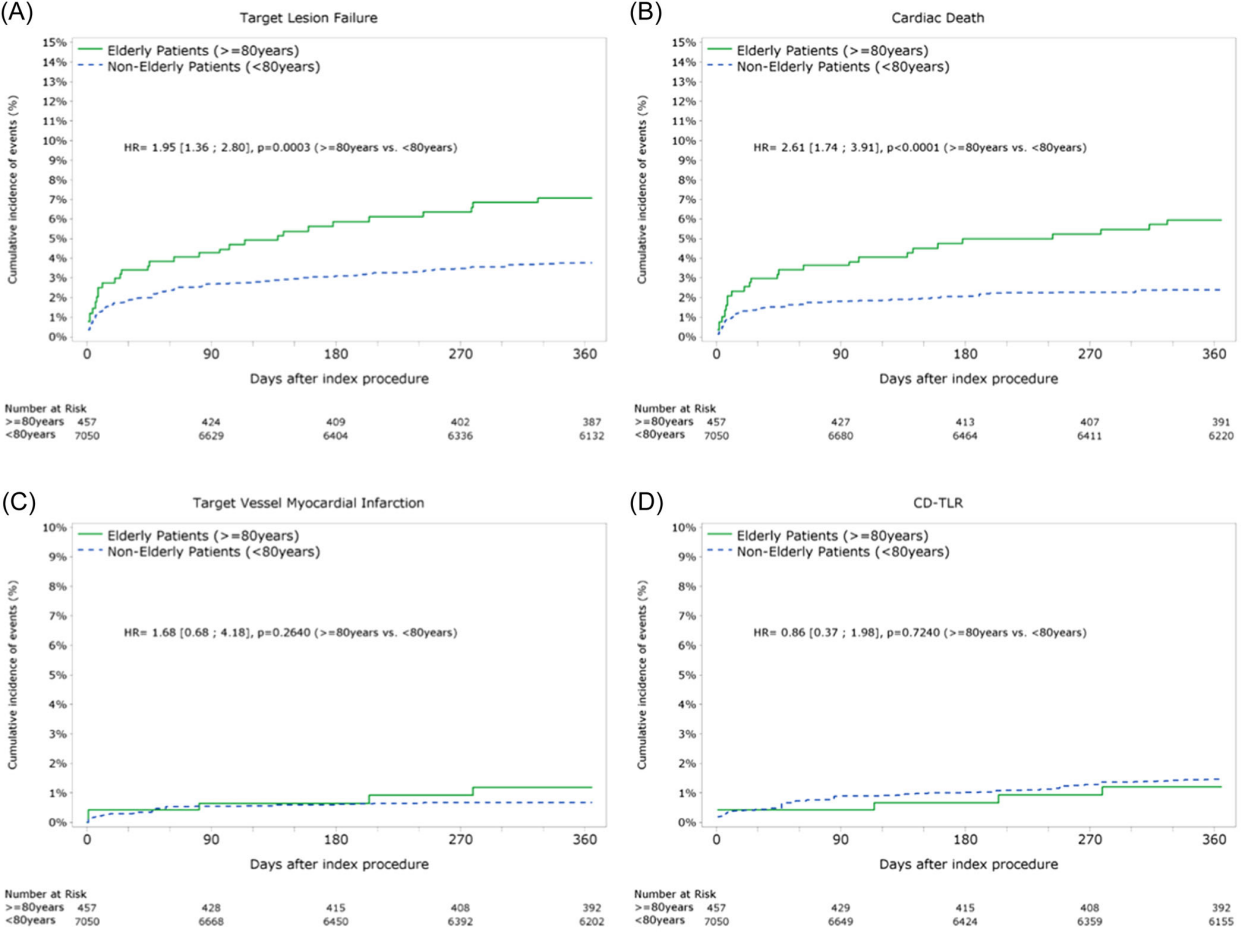
Kaplan–Meir cumulative incidence of events curves after inverse‐weighted propensity score adjustment. HR, hazard ratio.

After IPSW adjusted analysis, the incidence at 1 year for TLF (7.1% vs. 3.8%, *p* < .001), POCE (12.6% vs. 8.3%, *p* < .01), all death (10.1% vs. 3.6%, *p* < .0001), CD (6.0% vs. 2.4%, *p* < .0001), and bleeding (5.2% vs. 2.4%, *p* < .01) was higher for the elderly patients (Table 2, Figure 3). No difference in adjusted event rates was observed for all MI, TV‐MI, revascularization, and stent thrombosis.

## 4 DISCUSSION

We investigated this large, real‐world analysis of over 7000 all‐comer patients presenting with STEMI according to age. To our knowledge, this is amongst the largest analyses of PCI outcomes in all‐comers elderly patients presenting with STEMI who underwent PCI, and the largest with new‐generation thin strut DES. The crude and adjusted rates of the primary endpoint of TLF were significantly higher in the elderly group compared to the younger group. Age‐dependent outcomes such as all‐cause death, CD, POCE, and bleeding rates were also significantly higher in the elderly. However, PCI‐dependent variables such as the feasibility of the radial approach, and more importantly procedural outcomes, such as the number of MI, revascularizations, and stent thrombosis rates did not differ significantly.

Despite the growing need for PCI among the elderly, there is a paucity of data about the outcomes after PCI in this group. This group was frequently excluded from clinical trials, mainly because of concerns about the increased risk of adverse events and limited life expectancy.^11^, ^20^, ^23^, ^24^ More recently, new randomized trials were published, the SENIOR and XIMA trials that compared DES and BMS.^25^, ^26^ The latest published data from a patient‐level pooled analysis of the TWENTE trials included 671 elderly patients compared to 8533 younger patients, all treated with DES.^27^

In our study, the unadjusted rate of the primary endpoint of 1‐year TLF was significantly higher in the older age group (7.2% vs. 3.1%; *p* < .0001). This finding is mainly driven by the CD rate in the elderly group (6.1%), which is higher than the rates reported in the DES arm of the randomized XIMA, SENIOR, and TWENTE trials,^25^, ^26^, ^27^ but all those trials included mainly stable elderly patients and not only STEMI patients as our current analysis.

The elderly ACS‐2 multicenter trial included 1443 ACS patients aged >75 years (median age 80 years).^17^ As compared with those with non‐ST‐elevation ACS, STEMI patients (*n* = 592, 41%) had more favorable baseline risk factors, fewer prior cardiovascular events, and less severe coronary disease, but lower ejection fraction. At a median follow‐up of 12 months, 51 (8.6%) STEMI patients had died, versus 39 (4.6%) of non‐ST‐elevation ACS patients. After adjusting for sex, age, and previous myocardial infarction, elderly STEMI patients had worse survival and a higher risk of stroke compared with non‐ST‐elevation ACS patients after PCI. The mortality rates are similar to those reported in our current study.

The After Eighty study was an open‐label randomized controlled multicenter trial, that randomized 457 patients aged 80 years or older with NSTEMI or unstable angina to an invasive strategy or to a conservative strategy in Norway between 2010 and 2014.^18^ In this study, an invasive strategy was superior to a conservative strategy in the reduction of composite events. Efficacy of the invasive strategy was diluted with increasing age – after adjustment for creatinine and effect modification. The two strategies did not differ in terms of bleeding complications. Mortality was similar in both study arms 25% and 27% after an 18‐month follow‐up.

Among the 229 patients in the After Eighty study invasive group, the procedure was performed via the radial artery in 90%, 48% had three‐vessel disease or left main stenosis. Six patients (3%) underwent coronary artery bypass graft and PCI was performed in 107 patients (49%), with 57% treated with BMS, 37% DES, and 6% balloon angioplasty. Complications included one major PCI‐related bleeding (successfully treated) and 11 periprocedural myocardial infarctions.

As the results of our study show, the elderly group who underwent PCI had a more complex procedure with more interventions to the left main, smaller vessels, ostial lesions, and more often calcified lesions. Nonetheless, the transradial artery intervention (TRI) rates were high (>80%) both in the elderly and in the younger groups despite the higher complexity of PCI and higher comorbidities burden in the elderly group, a finding which is consistent with previous studies.^24^, ^25^ This finding emphasizes the feasibility as well as the high success rate of radial access also in fragile patients. A large Japanese registry has shown that TRI was an inverse‐independent predictor for both in‐hospital mortality and bleeding complications in both ACS and stable CAD cohorts.^20^

Safety is a major concern in elderly patients. Bleeding complications were more frequent in the elderly group compared to the younger group, with similar rates reported in studies using the new generation DES in the elderly.^15^, ^16^, ^17^, ^18^, ^19^, ^20^, ^21^, ^22^, ^23^, ^24^, ^25^, ^26^, ^27^ The higher proportion in the elderly group may be attributed to the higher prescription of anticoagulant medication as well as renal dysfunction found in this group.

Stent thrombosis is another aspect of postprocedural safety. The low rates of stent thrombosis in our trial are consistent with previous randomized trials with a polymer‐free, drug‐coated stent in patients at high bleeding risk and with a DES in patients in their eighties,^17^, ^18^, ^19^, ^20^, ^21^, ^22^, ^23^, ^24^, ^25^, ^26^, ^27^, ^28^ showing once more the advantages of using the new generation DES over older stents.

Life expectancy in any age group is different. A comparison of the 1‐year mortality rate between the groups has limitations, and the baseline life expectancy must be taken into consideration. The 1‐year mortality rate of 83‐year‐old adults (the mean age of patients in the elderly group) ranges between 4% and 5.7% for females and 6%–7.5% for males in the UK, France, and the USA, while the 1‐year mortality rate among 59‐year‐old adults is 0.5%–0.8% for females and 0.9%–1% for males.^29^, ^30^ Comparison of the mortality rate in our analysis (10.1% and 2.3%) to the expected life expectancy of the general population reveals that STEMI patients undergoing PCI, both elderly and controls, had a mortality rate that was twice higher than the expected mortality of the general age‐matched population.

In patients presenting with ACS, an invasive approach appears to demonstrate a better benefit–risk ratio compared to a conservative one, across different clinical patient complexity and multiple comorbidities. More powerful strategies of antithrombotic therapy for secondary prevention have been associated with increased bleeding events and have no benefit in terms of mortality reduction.^1^ Most evidence of ACS in the older patient focuses on age. However, age itself does not accurately reflect the patient's status, as other characteristics such as comorbidities and geriatric syndromes (frailty, disability, cognitive impairment, etc.) are the key determinants of a patient's health and vulnerability beyond age. In older patients with ACS, ethical considerations regarding management and treatment are common, especially when deciding invasive versus conservative treatment, type of drug therapy, and department of hospitalization. An interdisciplinary evaluation with geriatric assessment should always be considered to achieve a holistic approach and optimize any treatment on the basis of the underlying biological vulnerability.

### 4.1 Study limitations

Several potential limitations of this study should be noted. First, its observational nature may affect the way these results are translated into clinical grounds. Baseline characteristics were balanced by IPSW but unmeasured variables with a potential impact on outcomes could not be included. Second, the younger group included patients of a wide range of ages, some of them are close to the age of the elderly ≥80 years old. In addition, indices of frailty were not collected in this trial, as these parameters may vary widely among the elderly population, and, therefore, may affect the clinical outcomes. The findings of this study apply to a specific DES platform and cannot, therefore, be extrapolated to other bioabsorbable or durable polymer DES platforms.

## 5 CONCLUSION

In those presenting with STEMI, elderly patients had more comorbidities and a higher incidence of the composite endpoint TLF than younger patients. The all‐cause death, cardiac mortality, and bleeding rates at 1‐year were higher for elderly patients compared to patients younger than 80 years old. There was no difference in the incidence of PCI‐related outcomes such as recurrent revascularizations, MI, or stent thrombosis. These findings suggest that PCI in the elderly is relatively safe in the era of contemporary DES using this thin‐strut cobalt–chromium sirolimus‐eluting stent. However, even with contemporary DES technology and similar PCI‐related outcomes, elderly patients are still a higher risk population with higher rates of adverse events. This should also be taken into consideration before the decision regarding treatment strategies and the need for post‐STEMI follow‐up, in this age group.

## CONFLICT OF INTEREST

The authors declare no conflict of interest.

## Data Availability

Authors elect not to share data. Data are available on request due to privacy/ethical restrictions.

## 1

See Figures A1 and A2.

## References

[1] Morici N , De Servi S , De Luca L , et al. Management of acute coronary syndromes in older adults. Eur Heart J. 2022;43:1542‐1553. 34347065 10.1093/eurheartj/ehab391

[2] Renilla A , Barreiro M , Barriales V , Torres F , Alvarez P , Lambert JL . Management and risk factors for mortality in very elderly patients with acute myocardial infarction. Geriatr Gerontol Int. 2013;13:146‐151. 22672349 10.1111/j.1447-0594.2012.00876.x

[3] García‐Blas S , Cordero A , Diez‐Villanueva P , et al. Acute coronary syndrome in the older patient. J Clin Med. 2021;10:4132. 34575243 10.3390/jcm10184132 PMC8467899

[4] Parikh R , Chennareddy S , Debari V , et al. Percutaneous coronary interventions in nonagenarians: in‐hospital mortality and outcome at one‐year follow‐up. Clin Cardiol. 2009;32:E16‐E21. 10.1002/clc.20596 PMC6652868 20014200

[5] Sawant AC , Josey K , Plomondon ME , et al. Temporal trends, complications, and predictors of outcomes among nonagenarians undergoing percutaneous coronary intervention: insights from the veterans affairs clinical assessment, reporting, and tracking program. JACC Cardiovasc Interv. 2017;10:1295‐1303. 28683935 10.1016/j.jcin.2017.03.051

[6] Kim JY , Jeong MH , Choi YW , et al. Korea acute myocardial infarction registry investigators. temporal trends and in‐hospital outcomes of primary percutaneous coronary intervention in nonagenarians with ST‐segment elevation myocardial infarction. Korean J Intern Med. 2015;30:821‐828. 26552457 10.3904/kjim.2015.30.6.821 PMC4642011

[7] Mandawat A , Mandawat MK . Percutaneous coronary intervention after ST‐segment elevation myocardial infarction in nonagenarians: use rates and in‐hospital mortality. J Am Coll Cardiol. 2013;61:1207‐1208. 23375924 10.1016/j.jacc.2012.12.019

[8] Khan MA , Hashim MJ , Mustafa H , et al. Global epidemiology of ischemic heart disease: results from the global burden of disease study. Cureus. 2020;12(7):e9349. 32742886 10.7759/cureus.9349 PMC7384703

[9] Abizaid AS , Mintz GS , Abizaid A , et al. Influence of patient age on acute and late clinical outcome following Palmaz‐Schatz coronary stent implantation. Am J Cardiol. 2000;85:338‐343. 11078303 10.1016/s0002-9149(99)00743-2

[10] Gravina Taddei CF , Weintraub WS , Douglas JS, Jr. , et al. Influence of age on outcome after percutaneous transluminal coronary angioplasty. Am J Cardiol. 1999;84:245‐251. 10496430 10.1016/s0002-9149(99)00271-4

[11] Gurwitz JH , Goldberg RJ . Age‐based exclusions from cardiovascular clinical trials: implications for elderly individuals (and for all of us): comment on “the persistent exclusion of older patients from ongoing clinical trials regarding heart failure. Arch Intern Med. 2011;171:557‐558. 21444845 10.1001/archinternmed.2011.33

[12] Schoenenberger AW , Radovanovic D , Windecker S , et al. Temporal trends in the treatment and outcomes of elderly patients with acute coronary syndrome. Eur Heart J. 2016;37:1304‐1311. 26757786 10.1093/eurheartj/ehv698

[13] Zaman MJ , Stirling S , Shepstone L , et al. The association between older age and receipt of care and outcomes in patients with acute coronary syndromes: a cohort study of the myocardial ischaemia national audit project (MINAP). Eur Heart J. 2014;35:1551‐1558. 24644310 10.1093/eurheartj/ehu039

[14] Skolnick AH , Alexander KP , Chen AY , et al. Characteristics, management, and outcomes of 5,557 patients age > or =90 years with acute coronary syndromes: results from the CRUSADE initiative. J Am Coll Cardiol. 2007;49:1790‐1797. 17466230 10.1016/j.jacc.2007.01.066

[15] Puymirat E , Aissaoui N , Cayla G , et al. Changes in one‐year mortality in elderly patients admitted with acute myocardial infarction in relation with early management. Am J Med. 2017;130:555‐563. 28065766 10.1016/j.amjmed.2016.12.005

[16] Fernández‐Bergés D , Degano IR , Gonzalez Fernandez R , et al. Benefit of primary percutaneous coronary interventions in the elderly with ST segment elevation myocardial infarction. Open Heart. 2020;7(2):e001169. 32747454 10.1136/openhrt-2019-001169 PMC7402007

[17] Morici N , Savonitto S , Ferri LA , et al. Outcomes of elderly patients with ST‐elevation or non‐ST‐elevation acute coronary syndrome undergoing percutaneous coronary intervention. Am J Med. 2019;132:209‐216. 30447205 10.1016/j.amjmed.2018.10.027

[18] Tegn N , Abdelnoor M , Aaberge L , et al. Invasive versus conservative strategy in patients aged 80 years or older with non‐ST‐elevation myocardial infarction or unstable angina pectoris (After Eighty study): an open‐label randomised controlled trial. Lancet. 2016;387(10023):1057‐1065. 26794722 10.1016/S0140-6736(15)01166-6

[19] Biondi Zoccai G , Abbate A , D'Ascenzo F , et al. Percutaneous coronary intervention in nonagenarians: pros and cons. J Geriatr Cardiol. 2013;10:82‐90. 23610578 10.3969/j.issn.1671-5411.2013.01.013 PMC3627716

[20] Numasawa Y , Inohara T , Ishii H , et al. Comparison of outcomes after percutaneous coronary intervention in elderly patients, including 10 628 nonagenarians: insights from a Japanese nationwide registry (J‐PCI registry). J Am Heart Assoc. 2019;8:e011183. 10.1161/JAHA.118.011017 PMC6474917 30791799

[21] Miura T , Soga Y , Doijiri T , et al. Prevalence and clinical outcome of polyvascular atherosclerotic disease in patients undergoing coronary intervention. Circ J. 2013;77:89‐95. 23018634 10.1253/circj.cj-12-0535

[22] Codner P , Saada M , Sakhov O , et al. Proximal left anterior descending artery treatment using a bioresorbable polymer coating sirolimus‐eluting stent: real‐world outcomes from the multicenter prospective e‐ULTIMASTER registry. J Am Heart Assoc. 2019;8:e013786. 31787055 10.1161/JAHA.119.013786 PMC6912975

[23] Miura T , Miyashita Y , Motoki H , et al. Efficacy and safety of percutaneous coronary intervention for elderly patients in the second‐generation drug‐eluting stent era: the SHINANO registry. Angiology. 2017;68:688‐697. 27856669 10.1177/0003319716679341

[24] Kitabata H , Kubo T , Mori K , et al. Safety and efficacy outcomes of second‐generation everolimus‐eluting stents in octogenarians compared to non‐octogenarians. Cardiovasc Revasc Med. 2018;19(1 Pt A):12‐16. 10.1016/j.carrev.2017.05.022 28600019

[25] Varenne O , Cook S , Sideris G , et al. Drug‐eluting stents in elderly patients with coronary artery disease (SENIOR): a randomised single‐blind trial. Lancet. 2018;391(10115):41‐50. 29102362 10.1016/S0140-6736(17)32713-7

[26] De belder A , de la Torre Hernandez JM , Lopez‐Palop R , et al. A prospective randomized trial of everolimus‐eluting stents versus bare‐metal stents in octogenarians. J Am Coll Cardiol. 2014;63:1371‐1375. 24216285 10.1016/j.jacc.2013.10.053

[27] Ploumen EH , Buiten RA , Doggen C , et al. New‐generation drug‐eluting coronary stents in octogenarians: patient‐level pooled analysis from the TWENTE I‐IV trials. Am Heart J. 2020;228:109‐115. 32882569 10.1016/j.ahj.2020.07.003

[28] Morice MC , Talwar S , Gaemperli O , et al. Drug‐coated versus bare‐metal stents for elderly patients: a predefined sub‐study of the LEADERS FREE trial. Int J Cardiol. 2017;243:110‐115. 28579168 10.1016/j.ijcard.2017.04.079

[29] https://www.insee.fr/

[30] https://www.gov.uk/government/statistics/national-life-tables-life-expectancy-in-the-uk-2017-to-2019

